# Eukaryote hybrid genomes

**DOI:** 10.1371/journal.pgen.1008404

**Published:** 2019-11-27

**Authors:** Anna Runemark, Mario Vallejo-Marin, Joana I. Meier

**Affiliations:** 1 Department of Biology, Lund University, Lund, Sweden; 2 Biological and Environmental Sciences, University of Stirling, Stirling, Scotland, United Kingdom; 3 St John's College, Cambridge, Cambridge, United Kingdom; 4 Department of Zoology, University of Cambridge, Cambridge, United Kingdom

## Abstract

Interspecific hybridization is the process where closely related species mate and produce offspring with admixed genomes. The genomic revolution has shown that hybridization is common, and that it may represent an important source of novel variation. Although most interspecific hybrids are sterile or less fit than their parents, some may survive and reproduce, enabling the transfer of adaptive variants across the species boundary, and even result in the formation of novel evolutionary lineages. There are two main variants of hybrid species genomes: allopolyploid, which have one full chromosome set from each parent species, and homoploid, which are a mosaic of the parent species genomes with no increase in chromosome number. The establishment of hybrid species requires the development of reproductive isolation against parental species. Allopolyploid species often have strong intrinsic reproductive barriers due to differences in chromosome number, and homoploid hybrids can become reproductively isolated from the parent species through assortment of genetic incompatibilities. However, both types of hybrids can become further reproductively isolated, gaining extrinsic isolation barriers, by exploiting novel ecological niches, relative to their parents. Hybrids represent the merging of divergent genomes and thus face problems arising from incompatible combinations of genes. Thus hybrid genomes are highly dynamic and undergo rapid evolutionary change, including genome stabilization in which selection against incompatible combinations results in fixation of compatible ancestry block combinations within the hybrid species. The potential for rapid adaptation or speciation makes hybrid genomes a particularly exciting subject of in evolutionary biology. Here we summarize how introgressed alleles or hybrid species can establish and how the resulting hybrid genomes evolve.

## Background

Genetic exchange between species can impede the evolution of biodiversity because gene flow between diverging species counteracts their differentiation and hybridization between recently diverged species can lead to loss of genetic adaptations or species fusion[1]. Traditionally, zoologists have viewed interspecific hybridization as maladaptive behaviour[2] which can result in breaking up co-adapted gene complexes[3]. In contrast, plant biologists recognized early on that hybridization can sometimes be an important evolutionary force, contributing to increasing biodiversity[4][5]. Recently, evidence has been accumulating showing that hybridization is also an important evolutionary process in animals[1][6][7]. Interspecific hybridization can enrich the genetic diversity of introgressed taxa, lead to introgression of beneficial genetic variation or even generate new hybrid species[1]. Hybridization is now also known to contribute to the evolutionary potential in several textbook examples of adaptive radiation, including the *Geospiza* Galapagos finches[8], African cichlid fishes[9], *Heliconius* butterflies[10][11][12] and Hawaiian *Madiinae* tarweeds and silverswords[13]. Here we review the evolutionary outcomes of interspecific hybridization and the properties of genomes of hybrid genomes. Many of the discussed topics also apply to hybridization between different subspecies or populations of the same species, but here we focus on interspecific hybridization (referred to as hybridization in this review).

## Evolutionary outcomes of hybridization

**Fig 1:**
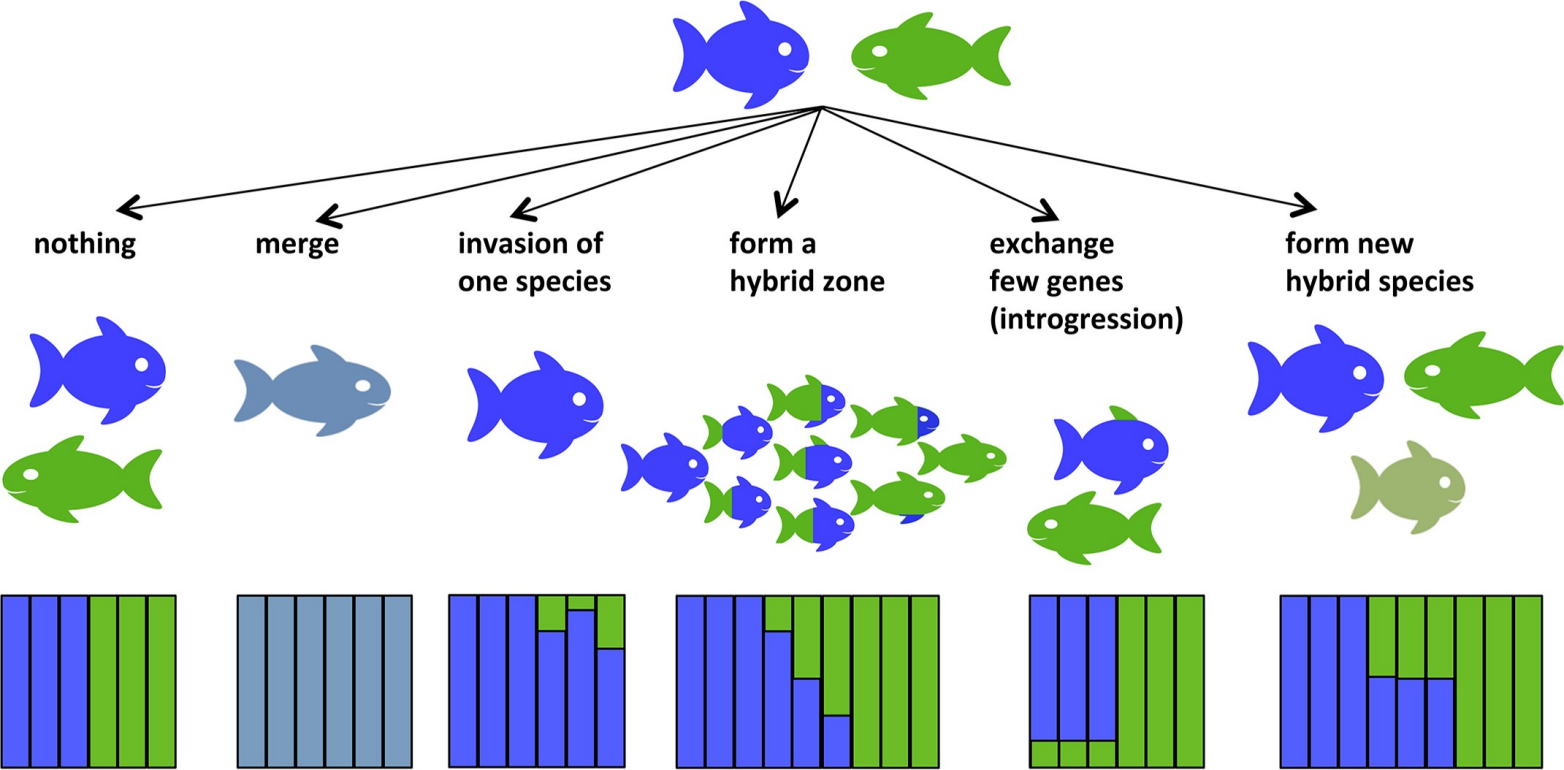
Potential evolutionary outcomes of hybridization. While most hybridization events are evolutionary dead ends, hybridization may also lead to speciation reversal where two taxa merge into one or form a hybrid zone between parapatric taxa. Alternatively, only one species may disappear through genetic swamping if introgression is highly asymmetrical. When one or few heterospecific alleles are advantageous these can also introgress into one of the parent species’ genomes through repeated backcrossing. Hybrids may also form novel lineages that are reproductively isolated from both parent taxa. The coloured fractions of the bars in the bar plots below show the relative proportion of the genome belonging to the blue and green parental lineages respectively. The grey bars represent a speciation reversal where differences are selected against. Finally, if hybridization leads to unfit offspring, it may reduce the fitness of the involved parental taxa due to wasted reproductive effort and may increase extinction risks for these.

There are several potential evolutionary outcomes of hybridization (Fig 1). If early generation hybrids are not viable or sterile, hybridization may reduce the reproductive success of the parent species[14][15]. This could potentially lead to reinforcement, selection to strengthen premating isolation[16] or if the species fail to evolve premating isolation, it could increase their extinction risk due to wasted reproductive effort[14]. If the fitness of early generation hybrids is non-zero and that of some later generation hybrids is as high or even higher than the fitness of one or both parent taxa, hybrids may displace the parent taxa and the hybridizing taxa may fuse (speciation reversal[17][18], Fig 1). If the fitness of early generation hybrids is reduced but non-zero, hybrid zones may emerge in the contact zone of the taxa[19]. If hybrids are fertile, hybridization may contribute novel variation through rare hybrids backcrosssing with parental species. Such introgressive hybridization may enable neutral or selectively beneficial alleles to be transferred across species boundaries even in species pairs that remain distinct despite occasional gene flow[20][21]. Hybrid fitness may vary with divergence time between the hybridizing taxa. This pattern has been shown for a variety of taxa including *Drosophila*,[22] birds[23] and fish[24]. Hybrid fitness may also differ with cross direction[25], between first generation and later generation hybrids[26], and among individuals within generations of the same cross-type[27][28]. In some cases hybrids may evolve into new hybrid species with reproductive isolation to both parent taxa[29][30]. Below we describe the evolutionary outcomes of hybridisation that result in persistent hybrid genomes.

## Adaptive introgression

When rare hybrids backcross with parent species alleles coding for traits that are beneficial for both parental species can be transferred across species boundaries, even if parent species remain distinct taxa. This process is referred to as adaptive introgression (a somewhat misleading term because backcrossing itself may not be adaptive, but some of the introgressed variants may be beneficial[1]). Simulations suggest that adaptive introgression is possible unless hybrid fitness is substantially reduced[31][32], or the adaptive loci are tightly linked to deleterious ones[33]. Examples of adaptive traits that have been transferred via introgression include an insecticide resistance gene that was transferred from *Anopheles gambiae* to *A. coluzzii*[21] and the red warning wing colouration trait in *Heliconius* butterflies that is under natural selection from predators and introgressed from *H. melpomene* to *H*. *timareta* [34] and other *Heliconius* species[20]. In the plant *Arabidopsis arenosa* some of the alleles conferring adaptation to drought and phytotoxic levels of metal have introgressed from *A. lyrata*[35]. Even in humans there is evidence for adaptive introgression of e.g. immunity alleles, skin pigmentation alleles and alleles conferring adaptation to high altitude environments from Neanderthal and Denisovans[36]. If traits important for species recognition or reproductive isolation introgress into a population of another species, the introgressed population may become reproductively isolated against other populations of the same species. Examples of this include *Heliconius* butterflies, where selective introgression of wing pattern genes between diverged lineages occurs (see e.g.[37]), and wing patterns contribute to reproductive isolation in some species pairs with low (e.g. between *H*. *t*. *florencia* and *H*. *t*. *linaresi*) and intermediate levels (e.g. *H*. *c*. *galanthus*/*H*. *pachinus*) of divergence[38]. See also Box 1.

**Box 1. Detecting and studying hybridization with genomic tools**

Many empirical case studies start with exploratory detection of putative hybrid taxa or individuals with genomic clustering approaches, such as STRUCTURE[142], ADMIXTURE[143] or fineSTRUCTURE[144]. These methods infer a user-specified number of genetic groups from the data and assign each individual to one or a mix of these groups. They can be applied to closely related taxa without having to preassign individuals to taxa and may thus be particularly useful in the study of closely related taxa or species complexes. However, uneven sampling of the parental taxa or different amounts of drift in the included taxa may lead to erroneous conclusions about evidence for hybridization[145]. If genomic data of multiple species is available, phylogenetic methods may be better suited to identify introgression. Introgressive hybridization leads to gene trees that are discordant from the species tree, whereby introgressed individuals are phylogenetically closer to the source of introgression than to their non-introgressed conspecifics. Such discordant gene trees can also arise by chance through incomplete lineage sorting, particularly if the species compared are still young. Therefore, discordant gene trees are only evidence of introgression if a gene tree produced by excess allele sharing between the hybridizing taxa is strongly overrepresented compared to alternative discordant gene trees. An entire suite of methods have been developed to detect such excess allele sharing between hybridizing taxa, including Patterson’s D statstics or ABBA-BABA tests[146][147][148] or f-statistics[149][150]. Modified versions of these tests can be used to infer introgressed genomic regions[151], the direction of gene flow[152][153] or the amount of gene flow[150]. For datasets with a large number of taxa it may be difficult to compute all possible test of hybridization. In such cases, graph construction methods may be better suited[154][155][156]. These methods reconstruct complex phylogenetic models with hybridization that best fit the genetic relationships among the sampled taxa and provide estimates for drift and introgression. Other phylogenetic network methods that account for incomplete lineage sorting and hybridization may also help[157][158]. Methods based on linkage disequilibrium decay or methods inferring ancestry tracts can be used to date recent admixture or introgression events as over time ancestry tracts are continuously broken down by recombination[155][159][160][161][162]. With increasing genome stabilization, individuals should vary less in local ancestry. Levels of genome stabilization can thus be assessed by computing the ancestry proportions (e.g. with f_d_[151]) in genomic windows and testing if these correlate across individuals. Additionally, if hybridization is still ongoing, ancestry proportions should vary across individuals and in space. A different approach is to use demographic modelling to find the simplification of the evolutionary history of the studied taxa[163]. Demographic modelling should only be applied to small sets of taxa because with increasing number of taxa model complexity increases and the number of model parameters such as timing, amounts and direction of gene flow, and population sizes and split times can quickly become too high. The fit of the demographic models to the data can be assessed with the site frequency spectrum[164][165] or with summary statistics in an Approximate Bayesian Computation framework[166]. It is also possible to gain more power by combining information from linkage disequilibrium decay patterns and the allele frequency spectrum[167].

## What is a hybrid species?

One of the potential evolutionary outcomes of hybridisation is the establishment of a novel, reproductively isolated lineage, i.e., hybrid speciation[1][29]. A hybrid species has an admixed genome and forms stable genetically distinct populations[29]. Some researchers argue that evidence of a hybridization-derived basis for reproductive isolation should be an additional defining criterion for hybrid speciation[39] but see[40]. This stricter definition includes polyploid hybrid taxa but only encompasses a handful of well studied cases of homoploid hybrid speciation, e.g. *Heliconius heurippa*[10][11][12], *Passer italiae*[28], and three *Helianthus* sunflower species[41] because for most suggested examples of homoploid hybrid speciation, the genetic basis of reproductive isolation is still unknown[39].

Hybrid species can occupy an ecological niche different to those of the parents and may be isolated from the parent species primarily through pre-mating barriers (hybrid speciation with external barriers, c.f. [42]). Hybrid species may also be reproductively isolated from the parent species through sorting of incompatibilities leading to new combinations of parental alleles that are incompatible with both parent species but compatible within the hybrid taxon (recombinational hybrid speciation)[29]. A recombinational hybrid taxon typically also has a substantial proportion of the genome derived from the donor of introgressed material, although variation exists both between taxa and within lineages of hybrid taxa (see e.g.[43][44]).

## Homoploid and polyploid hybrid speciation

**Fig 2:**
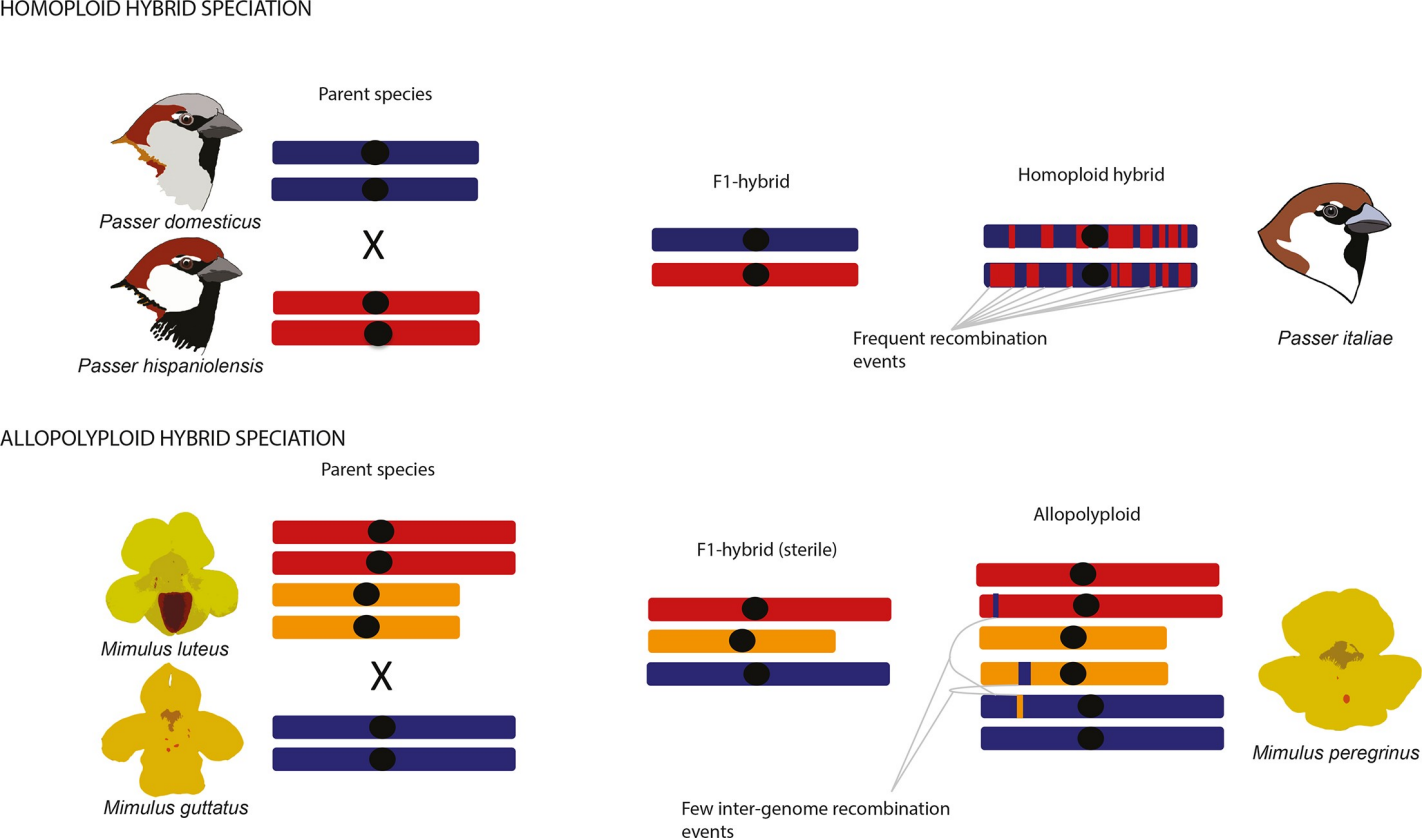
Schematic representation of homoploid and allopolyploid hybrid speciation. As an example of a homoploid hybrid genome we present a schematic figure of the mosaic genome of the Italian sparrow which is a hybrid resulting from the anthropogenic house sparrow *P. domesticus* which spread across the Mediterranean with agriculture and encountered and hybridized with local populations of Spanish sparrow *P. hispaniolensis* [44,47,85]. As allopolyploid example we use the monkeyflower *Mimulus peregrinus*, an allohexaploid species that has evolved independently at least twice and which involves an intermediate, sexually-sterile but clonally vigorous F1 hybrid [115]. Sterile F1 hybrids have given rise to allopolyploids in other taxa (e.g. *Spartina* and *Senecio*), but allopolyploids can also form via fertile intermediate hybrids (e.g. *Tragopogon*).

In general, hybrid species can arise from two major types of hybrid speciation, defined by whether the speciation event is associated with genome duplication (polyploidy) or not. Homoploid hybrid speciation is defined as the evolution of a new hybrid species with reproductive isolation to both parent taxa without change of ploidy, i.e. number of chromosome sets (Fig 2)[1]. The genomes of homoploid hybrid species are mosaics of the parent genomes as ancestry tracts from the parent species are broken up by recombination[40][41][45][46][47][48][49]. In the case of polyploid hybrid speciation, hybridisation is associated with genome duplication, resulting in an allopolyploid with increased ploidy compared to their parental taxa (Fig 2). In contrast to allopolyploids, autopolyploids are characterised by genome duplication within the same species and are thus not discussed further in the context of this review. Allopolyploid speciation is more common in plants than in animals[50]. Polyploid hybrids can be instantly isolated from their parental species through chromosome number differences[50].

## Reproductive isolation against parental species

Sufficient reproductive isolation from both parental species is required for the successful establishment of a hybrid species[1][39][51]. Reproductive isolation against parent species is harder to achieve for homoploid hybrids where karyotype differences do not contribute to intrinsic isolation. Reproductive isolation between a hybrid species and its parental species can arise from a variety of reproductive barriers either before or after fertilization (prezygotic or postzygotic, respectively), which may themselves be dependent or independent of environmental conditions (extrinsic or intrinsic barriers, respectively)[52]. For example, intrinsic postzygotic barriers cause hybrid inviability or sterility regardless of the environment in which they occur, while extrinsic postzygotic barriers result in hybrids of low fitness due to maladaptation to specific environments[30].

Prezygotic intrinsic and extrinsic differences have also been shown to be important in isolating hybrids from their parent species. In plants, pollinator-mediated isolation resulting from changes in floral characteristics may be an important extrinsic prezygotic ecological barrier[53][54][55][56]. Strong extrinsic pre-zygotic barriers have been shown to isolate the hybrid species *Senecio eboracensis* from its parent species, where hybrids are virtually absent in the wild, although a fraction of hybrid offspring are fertile in lab experiments[57]. Lowe & Abbott conclude that selfing, timing of flowering and characters involved in pollinator attraction likely contribute to this external isolation[57]. Prezygotic mate preference driven isolation generated from intrinsic assortative mating between hybrids has also been reported in several taxa. In African cichlid fish, experimental hybrids displayed combinations of parental traits and preferences which resulted in hybrids predominantly mating with other hybrids[58]. A similar pattern was found in *Geospiza* Galapagos finches where a specific hybrid song resulted from the transgressive beak morphology[8], and hybrid *Heliconius* butterflies preferred the hybrid wing patterning over that of both parental species[12]. Intrinsic differences in habitat use[59] or in phenology[60] may result in some degree of reproductive isolation against parental species if mating is time and habitat-specific. For example the apple host race in *Rhagoletis pomonella* maggot flies evolved after introgression of diapause related genes from Mexican altiplano flies that allowed a switch from the ancestral host hawthorne to the later flowering apple [61][62] and isolated the two host races via allochronic intrinsic pre-zygotic isolation. In *Xiphophorus* swordtail fish strong ancestry-assortative mating maintained a hybrid genetic cluster separate for 25 generations, but disappeared under manipulated conditions[63]. Hence, prezygotic reproductive barriers to gene flow may be environment dependent.

Postzygotic isolating barriers have also been shown to be important in a variety of hybrid lineages. Work on *Helianthus* sunflowers has revealed that intrinsic postzygotic can cause reproductive isolation against the parent species. The postzygotic barriers consist in pre-existing structural differences[47][64], in combination with hybridization induced structural differences[47]. Sorting of incompatibilities between parent species, where one subset of these isolates the hybrid taxon against one parent and a different subset isolates it against the other parent, has resulted in intrinsic postzygotic isolation between the Italian sparrow *Passer italiae* and its parent species[28]. Simulation studies show that the likelihood of hybrid speciation through this mechanism depends on the divergence time between parent species[65], the population size of the hybrid species[66], the nature of selection acting on hybrids, and linkage among incompatibilities to each other and to adaptive variants[67]. Extrinsic ecological barriers against parent species may arise as by-products of ecological differentiation if mating is time and/or habitat specific. Hybrid species have been shown to adapt to novel ecological niches through transgressive phenotypes[59], or through novel combinations of ecological traits from the parent species[68], and ecological selection against parent-hybrid cross phenotypes would result in extrinsic postzygotic isolation.

## Stabilization of hybrid genomes

Hybridization can have many different outcomes. Hybrid speciation results in reproductive isolation against both parent species and genomes that evolve independently from those of the parent species. Introgressive hybridization can transfer important novel variants into genomes of a species that remains distinct from the other taxa in spite of occasional gene flow. Here we refer to both types of hybridization-derived genomes as persistent hybrid genomes. Following initial hybridization, introgression tracts, the genetic blocks inherited from each parent species, are broken down with successive generations and recombination events. Recombination is more frequent in homoploid hybrid genomes than in allopolyploid hybrid genomes. In allopolyploids, recombination can destabilize the karyotype and lead to aberrant meiotic behaviour and reduced fertility, but may also generate novel gene combinations and advantageous phenotypic traits [69] as in homoploid hybrids. Once hybridization between the hybrid taxon and its parent taxa ceases, different ancestry blocks or introgression tracts may become fixed, a process referred to as "genome stabilization"[45]. Some introgression tracts are removed by selection against incompatibilities and others are fixed. Theoretical models on hybrid zones suggest that the breakdown of ancestry blocks through recombination is suppressed near genes conferring reproductive isolation due to lower fitness of recombinant hybrids[70]. The strength of the suppression is affected by the form of selection, dominance, and whether the locus is situated on an autosome or sex chromosome[70]. The time to genome stabilization is variable. Fixation of ancestry blocks was found to be rapid in experimental hybrid *Helianthus* sunflower species genomes[71], and the genome stabilization of hybrid sunflower species is estimated to take hundreds of generations[45]. In *Zymoseptoria* fungi genomes were stabilized within ca. 400 generations,[72] and hybrid *Xiphophorus* swordtail genomes[73] genome stabilization was achieved after ca. to 2500 generations. Few Neanderthal regions have fixed in human genomes during the ca. 2000 generations after hybridization[74], and segregating incompatibilities are present in the hybrid Italian sparrow approximately 5000 generations after the initial hybridization event[75].

**Fig 3:**
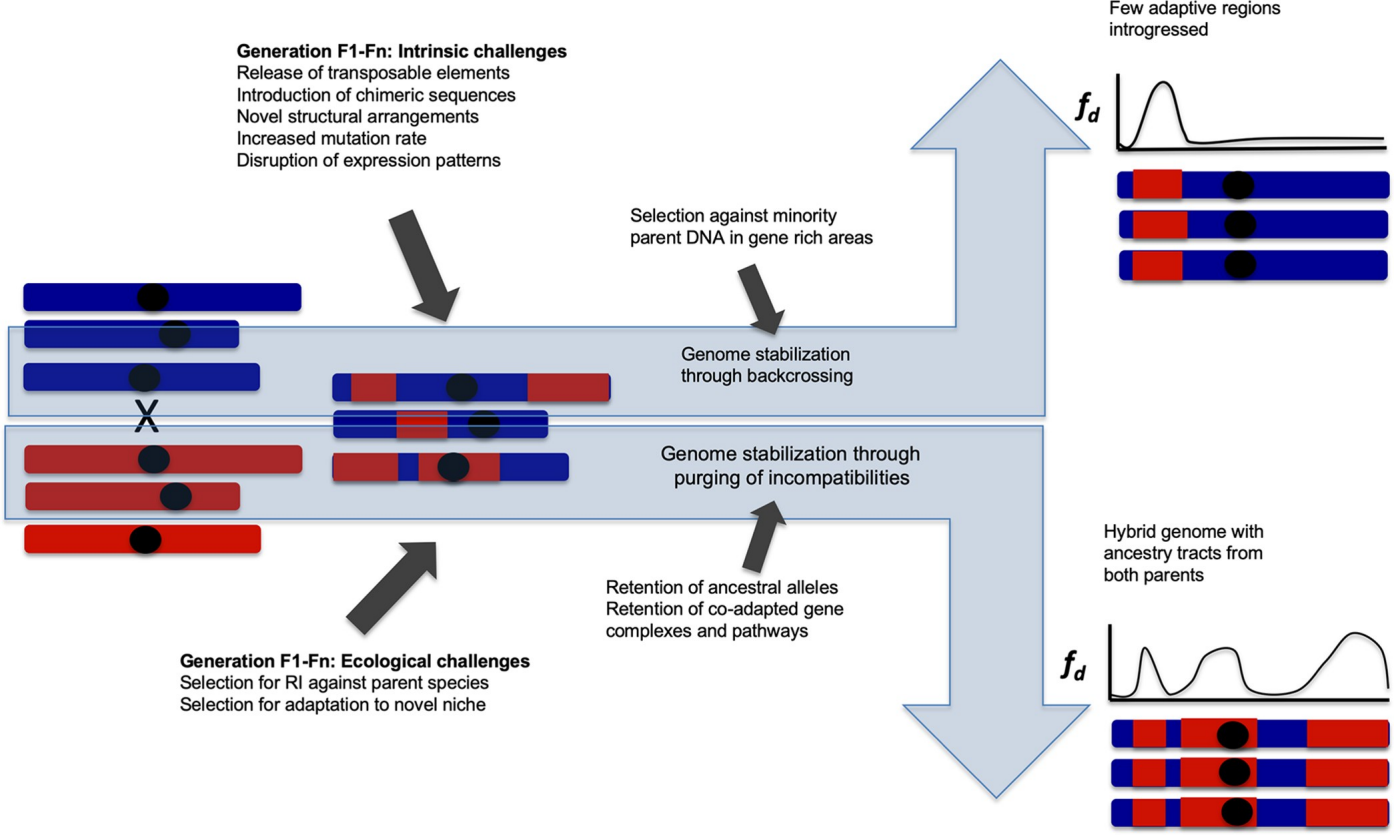
The process of genome stabilization during hybrid speciation and introgression. Both ecological selection pressures and selection to avoid intrinsic incompatibilities mould hybrid genomes. Depending on the balance between beneficial alleles and incompatibilities hybridisation can result either in an admixed taxon that is reproductively isolated from both parent taxa, or local introgression into a taxon that remains distinct in spite of occasional gene flow. RI abbreviates reproductive isolation. Fd is a measure of introgression and is estimated between a hybrid population and the red parent species, and the haplotypes illustrate example individuals in these populations.

Given time, genetic drift will eventually stochastically fix blocks derived from the two parent species in finite isolated hybrid populations[45]. Selection against incompatibility loci may accelerate the process of fixation of parental alleles as hybrids that possess alleles that are less likely to cause incompatibility will have higher fitness and favourable alleles will spread in the population. Fixation of recessive weakly deleterious alleles in the parent taxa may, however, also result in hybrids retaining both parental alleles: because hybrids with haplotypes from both parents are not homozygous for any weakly deleterious alleles, they have higher fitness than hybrids with only one parental haplotype. This associative overdominance[76][77], may slow down the process of fixation of parental alleles through favouring retention of both parental haplotypes. The effect of associative overdominance is strongest in low recombination regions, including inversions[78]. The balance between alleles and allelic combinations providing favourable phenotypic characters and the strength of selection against incompatibilities determine what introgression tracts will be inherited from which parent species upon hybridization (Fig 3)[21][79][80]. An insecticide resistance region was retained following a hybridization event in *Anopheles coluzzi*[21], suggesting a role for selection in maintaining favourable introgressed regions. The local recombination rate is important for the likelihood of introgression because in the case of widespread incompatibilities, introgressed alleles are more likely to recombine away from incompatibilities in high recombination regions. This pattern has been detected in monkeyflowers *Mimulus*[81], in *Mus domesticus* house mice[82], in *Heliconius* butterflies[80] and in *Xiphophorus* swordtail fish[43].

Genome-wide incompatibilities have been identified in *Xipophorous* fish,[83] chimeric genes and mutations of orthologous genes cause incompatibilities in early generation experimental *Cyprinidae* goldfish—carp hybrids[84] and mito-nuclear incompatibilies are found to have a key role e.g. in Italian sparrows[49][85], fungus[86] and cyto-nuclear incompatibilities in *Mimulus* plants[87]. Evidence from altered expression patterns in synthetic hybrids and missing gene combinations in a hybrid species also suggest that DNA-repair[49][84][88] and genes involved in mutagenesis and cancer related pathways[84] may cause incompatibilities in hybrids. Genome formation in hybrid species is shaped by selection against incompatible combinations[43][73][79].

## Altered genome properties in hybrid taxa

The hybrid origin may affect genome structure and properties. It has been shown to increase mutation rates[52][89][90], to activate transposable elements[91][92][93], and to induce chromosomal rearrangements[94][95]. Increased transposon activation, as proposed in McClintock's ‘genomic shock’ theory, could result in alterations to gene expression[95]. Transposable elements may, in addition to altering gene products if inserted into a gene, also alter promoter activity for genes if inserted upstream of the coding regions, or may induce gene silencing as a result of gene disruption[96][97]. For allopolyploid genomes chromosomal rearrangements may result from the genomic shock induced by hybridisation, with more distantly related species being more prone to genome reorganisations e.g. in *Nicotiana*[98]. Chromosomal rearrangements resulting from either genomic shock or recombination events between non-homologous subgenomes may cause genome sizes to either increase or decrease[99]. Both increases and decreases were found in the *Nicotiana* genus, and were not related to the age since hybridization[100].

Following genome duplication in allopolyploids, the genome goes through diploidization, which is a process in which the genome is rearranged to act as a meiotic diploid [101][102]. After such diploidization, much of the genome is lost due to genome fractionation, the loss-of-function of one or the other of the newly duplicated genes[102][103]. In a meta analysis, Sankoff and collaborators found evidence consistent with reduction-resistant pairs and a concentration of functional genes on a single chromosome and suggest that the reduction process partly is constrained[103].

A related allopolyploid specific phenomenon is subgenome dominance. For example, in the octoploid *Fragaria* strawberry, one of the four subgenomes is dominant and has significantly greater gene content, more frequently has its genes expressed, and exchanges between homologous chromosomes are biased in favour of this subgenome, as compared with the other subgenomes[104]. This study also showed that certain traits, e.g. disease-resistance, are controlled by the dominant subgenome to a high extent[104]. A proposed mechanism of how subgenome dominance arises, suggests that relative dominance is related to the density of transposable elements in each subgenome. Subgenomes with higher transposable element density tend to behave submissively relative to the other subgenomes when brought together in the allopolyploid genome[102][105]. Interestingly, subgenome dominance can arise immediately in allopolyploids, as shown in synthetic and recently evolved monkeyflowers[105].

In addition to these changes to genome structure and properties, studies of allopolyploid rice and whitefish suggest that patterns of gene expression may be disrupted in hybrid species[106][107]. Studies of synthetic and natural allopolyploids of *Tragopogon miscellus* show that gene expression is less strictly regulated directly after hybridization, and that novel patterns of expression emerge and are stabilized during 40 generations[108]. While expression variation in miRNAs alters gene expression and affects growth in the natural allopolyploid *Arabidopsis suecica* and experimental lineages, inheritance of siRNAs is stable and maintains chromatin and genome stability[109], potentially buffering against a transcriptomic shock.

## What factors influence the likelihood of formation of persistent hybrid genomes?

Whereas hybridization is required for the generation of persistent hybrid genomes, it is not sufficient. For the persistence of hybrid genomes in hybrid species they need to be sufficiently reproductively isolated from their parent species to avoid species fusion. Selection on introgressed variants allows the persistence of hybrid genomes in introgressed lineages. Frequency of hybridization, viability of hybrids, and the ease at which reproductive isolation against the parent species arises or strength of selection to maintain introgressed regions are hence factors influencing the rate of formation of stable hybrid lineages.

Few general conclusions about the relative prevalence of hybridization can be drawn, as sampling is not evenly distributed across the tree of life, even if there is evidence for hybridization in an increasing number of taxa. One pattern that emerges is that hybridization is more frequent in plants where it occurs in 25% of the species, whereas it only occurs in 10% of animal species[110]. Most plants, as well as many groups of animals, lack heteromorphic sex chromosomes[111]. The absence of heteromorphic sex chromosomes results in slower accumulation of reproductive isolation[112][113], and may hence enable hybridization between phylogenetically more distant taxa. Haldane's rule[114] states that”when F1 offspring of two different animal races one sex is absent, rare, or sterile, that sex is the heterozygous sex”. Empirical evidence supports a role for heteromorphic sex chromosomes in hybrid sterility and inviability. A closely related observation is the large X effect stating that there is a disproportionate contribution of the X/Z-chromosome in fitness reduction of heterogametic hybrids[22]. These patterns likely arise as recessive alleles with deleterious effects in hybrids have stronger impacts on the heterogametic than the homogametic sex, due to hemizygous expression[115]. In taxa with well-differentiated sex chromosomes, Haldane’s rule has shown to be close to universal, and heteromorphic sex chromosomes show reduced introgression on the X in XY (see [116] for a review). In line with a role for heteromorphic sex chromosomes in constraining hybrid genome formation, elevated differentiation on sex chromosomes has been observed in both ZW and XY systems[117]. This pattern may reflect the lower effective population sizes and higher susceptibility to drift on the sex chromosomes[118], the elevated frequency of loci involved in reproductive isolation[119] and/or the heightened conflict on sex chromosomes[120]. Findings of selection for uniparental inheritance of e.g. mitonuclear loci residing on the Z chromosome in hybrid Italian sparrows[49] is consistent with compatible sex chromosomes being important for the formation of a viable hybrid genomes.

There are also several ecological factors that affect the probability of hybridization. Generally, hybridization is more frequently observed in species with external fertilization including plants but also fishes, than in internally fertilized clades[4]. In plants, high rates of selfing in some species may prevent hybridization, and breeding system may also affect the frequency of heterospecific pollen transfer[121][122]. In fungi, hybrids can be generated by ameiotic fusion of cells or hyphae[123] in addition to mechanisms available to plants and animals. Such fusion of vegetative cells and subsequent parasexual mating with mitotic crossover may generate recombined hybrid cells[123].

For hybrid species to evolve, reproductive isolation against the parental species is required. The ease by which such reproductive isolation arises is thus also important for the rate at which stable hybrid species arise. Polyploidisation and asexual reproduction are both mechanisms that result in instantaneous isolation and may increase the rate of hybrid lineage formation. The ability to self-pollinate may also act in favour of stabilising allopolyploid taxa by providing a compatible mate (itself) in the early stages of allopolyploid speciation when rare cytotypes are at a reproductive disadvantage due to inter-cytotype mating[124]. Selfing is also expected to increase the likelihood of establishment for homoploid hybrids according to a modelling study[125], and the higher probability of selfing may contribute to the higher frequency of hybrid species in plants. Fungal hybridization may result in asexual hybrid species, as *Epichloe* fungi where hybrids species are asexual while nonhybrids include both asexual and sexual species[126]. Hybridization between strongly divergent animal taxa may also generate asexual hybrid species, as shown e.g. in the European spined loaches, *Cobitis*[127], and most if not all asexual vertebrate species are of hybrid origin[128]. Interestingly, Arctic floras harbour an unusually high proportion of allopolyploid plants[129], suggesting that these hybrid taxa could have an advantage in extreme environments, potentially through reducing the negative effects of inbreeding. Hence, both genomic and ecological properties may affect the probability of hybrid species formation.

For introgressed taxa, the strength of selection on introgressed variants decides whether introgressed sections will spread in the population and stable introgressed genomes will be formed. Strong selection for insecticide resistance has been shown to increase introgression of an *Anopheles gambiae* resistance allele into *A*. *coluzzi* malaria mosquitoes[130]. In *Heliconius* butterflies, strong selection on having the locally abundant wing colour patterns repeatedly led to fixation of alleles that introgressed from locally adapted butterflies into newly colonizing species or subspecies[34]. Chances of fixation of beneficial introgressed variants depend on the type and strength of selection on the introgressed variant and linkage with other introgressed variants that are selected against.

## What genes or genomic regions are affected by hybridization?

Genetic exchange can occur between populations or incipient species diverging in geographical proximity or between divergent taxa that come into secondary contact. Hybridization between more diverged lineages is expected to have a greater potential to contribute beneficial alleles or generate novelty than hybridization between less diverged populations because more divergent alleles are combined, and are thus more likely to have a large fitness effect, to generate transgressive phenotypes[131]. Hybridization between more diverged lineages is also more likely to generate incompatible allele combinations, reducing initial hybrid fitness[132] but potentially also contributing to hybrid speciation if they are sorted reciprocally as described above[131]. An intermediate genetic distance may thus be most condusive to hybrid speciation[131]. Experimental lab crosses support this hypothesis[65].

The proportion of the genome that is inherited from the recipient of introgressed material varies strongly among and within species. After the initial hybridization event the representation is 50% in many polyploid taxa, although parental gene copies are successively lost and might bias the contribution to one majority parent genome[133]. Relatively equal parental contributions are also found in some homoploid hybrid species[48] but in other cases they are highly unequal such as in some *Heliconius* species[134]. The majority ancestry may even be that from the donor of introgressed material, as was shown for *Anopheles gambiae* mosquitoes.[135] Interestingly there may also be variation in parental contribution within a hybrid species. In both swordtail fish and Italian sparrows there are populations which differ strongly in what proportions of the parent genomes they have inherited[43][44].

Patterns of introgression can vary strongly across the genome, even over short chromosomal distances. Examples of adaptive introgression of well defined regions, include an inversed region containing genes involved in insecticide resistance[21] and introgression of a divergent, inverted chromosomal segment has resulted in a”super gene” that encodes mimicry polymorphism in the butterfly *Heliconius numata*[136]. These findings are consistent with models suggesting that genomic rearrangements are important for the coupling of locally adaptive loci[137]. Genes and genomic regions that are adaptive may be readily introgressed between species e.g. in hybrid zones if they are not linked to incompatibility loci. This often referred to semi-permeable species boundaries[19][138][139], and examples include e.g. genes involved in olfaction that are introgressed across a *Mus musculus* and *M*. *domesticus* hybrid zone[140]. In hybrid zones with mainly permeable species boundaries, patterns of introgressed regions enable deducing what genomic regions are involved in incompatibilities and reproductive isolation [141].

## Conclusions and future directions

Hybridization is a common phenomenon with a wide range of consequences. These include both the formation of novel hybrid species, which are reproductively isolated from their parent species and where the admixed genomes undergo independent evolution, and introgression of adaptive variants across species boundaries in species that remain distinct in spite of occasional gene flow. The divergent genetic material in admixed genomes of hybrid taxa enables adaptation to novel environments and niches. When the divergent genomes of two species come together, incompatible combinations may reduce fitness. As hybrid genomes are frequently observed, the advantage of novel adaptive trait combinations can sometimes override potential negative effects from incompatibilities and enable hybrid lineages to purge these incompatibilities during the process of genome stabilization.

While the last decades have provided ample evidence for that hybrid genome formation is common and contributes novel species and enables adaptation, many questions remain. How long does it take for a hybrid genome to stabilize and why is there variation in time to genome stabilization[45][73]? To what extent are hybrid genomes shaped by selection for compatibility? Is there a tendency for reversal towards one parent species during genome stabilization in homoploid hybrids? Does donor ancestry typically remain primarily in high recombination tracts [43] or are there generally stable solutions with high contributions from both parent species across the genome [49]? What are the relative effects of hybridization vs. polyploidization in generating new phenotypes during allopolyploid speciation? Does time to stabilization differ between homoploid and allopolyploid hybrid taxa? Are most orthologous genes lost over time in allopolyploid hybrids leaving only the ones where it is advantageous to have both as double copies [99][133]? Does genome size in allopolyploids vary predictably with taxon age or does this vary as in *Nicotiana*[100]? Hybrid genomes are important components of biodiversity and hybrid origin may spur adaptation. Future investigations into the properties of hybrid genomes will improve our understanding of the potential of hybridization to produce novel adaptive variation.

## Supporting information

S1 Text Version history of the text file. (XML)

S2 Text Peer reviews and response to reviews. (XML)
